# Has COVID-19 Changed the Hedge Effectiveness of Bitcoin?

**DOI:** 10.3389/fpubh.2021.704900

**Published:** 2021-07-27

**Authors:** Yinpeng Zhang, Panpan Zhu, Yingying Xu

**Affiliations:** ^1^College of Economics, Shenzhen University, Shenzhen, China; ^2^School of Economics, Beijing Technology and Business University, Beijing, China; ^3^School of Economics and Management, University of Science and Technology Beijing, Beijing, China

**Keywords:** COVID-19, bitcoin, dynamic correlation, volatility spillover, hedging effectiveness

## Abstract

The Bitcoin market has become a research hotspot after the outbreak of Covid-19. In this paper, we focus on the relationships between the Bitcoin spot and futures. Specifically, we adopt the vector autoregression-dynamic correlation coefficient-generalized autoregressive conditional heteroskedasticity (VAR-DCC-GARCH) model and vector autoregression-Baba, Engle, Kraft, and Kroner-generalized autoregressive conditional heteroskedasticity (VAR-BEKK-GARCH) models and calculate the hedging effectiveness (HE) value to investigate the dynamic correlation and volatility spillover and assess the risk reduction of the Bitcoin futures to spot. The empirical results show that the Bitcoin spot and futures markets are highly connected; second, there exists a bi-directional volatility spillover between the spot and futures market; third, the HE value is equal to 0.6446, which indicates that Bitcoin futures can indeed hedge the risks in the Bitcoin spot market. Furthermore, we update the data to the post-Covid-19 period to do the robustness checks. The results do not change our conclusion that Bitcoin futures can hedge the risks in the Bitcoin spot market, and besides, the post-Covid-19 results indicate that the hedging ability of Bitcoin futures increased. Finally, we test whether the gold futures can be used as a Bitcoin spot market hedge, and we further control other cryptocurrencies to illustrate the hedging ability of the Bitcoin futures to the Bitcoin spot. Overall, the empirical results in this paper will surely benefit the related investors in the Bitcoin market.

## Introduction

The global outbreak of Covid-19 has led to over 87.6 million cumulative confirmed cases, with over 1.9 million deaths as of January 2021, according to the official dataset by the World Health Organization (WHO). The disease is highly contagious, and, consequently, the global healthcare systems, the real economy, and the financial sphere are severely affected (1, 2). What is worse, according to Hanif et al. (3), the economic and financial consequences of the Covid-19 pandemic exceed those of the 2008 global financial crisis (GFC). The Covid-19 pandemic outbreak has paralyzed both the domestic and international economic activity and financial markets in countries. Thus, major countries have implemented the expansionary monetary policy to help the economy to survive from the Covid-19. Despite the intention is to help the economy recover, monetary policies have inevitably pushed up asset prices. Among all the assets that are rising in price, Bitcoin, one of the most commonly known cryptocurrencies, is of particular interest, as the price increases are very substantial.

**Figure 1:**
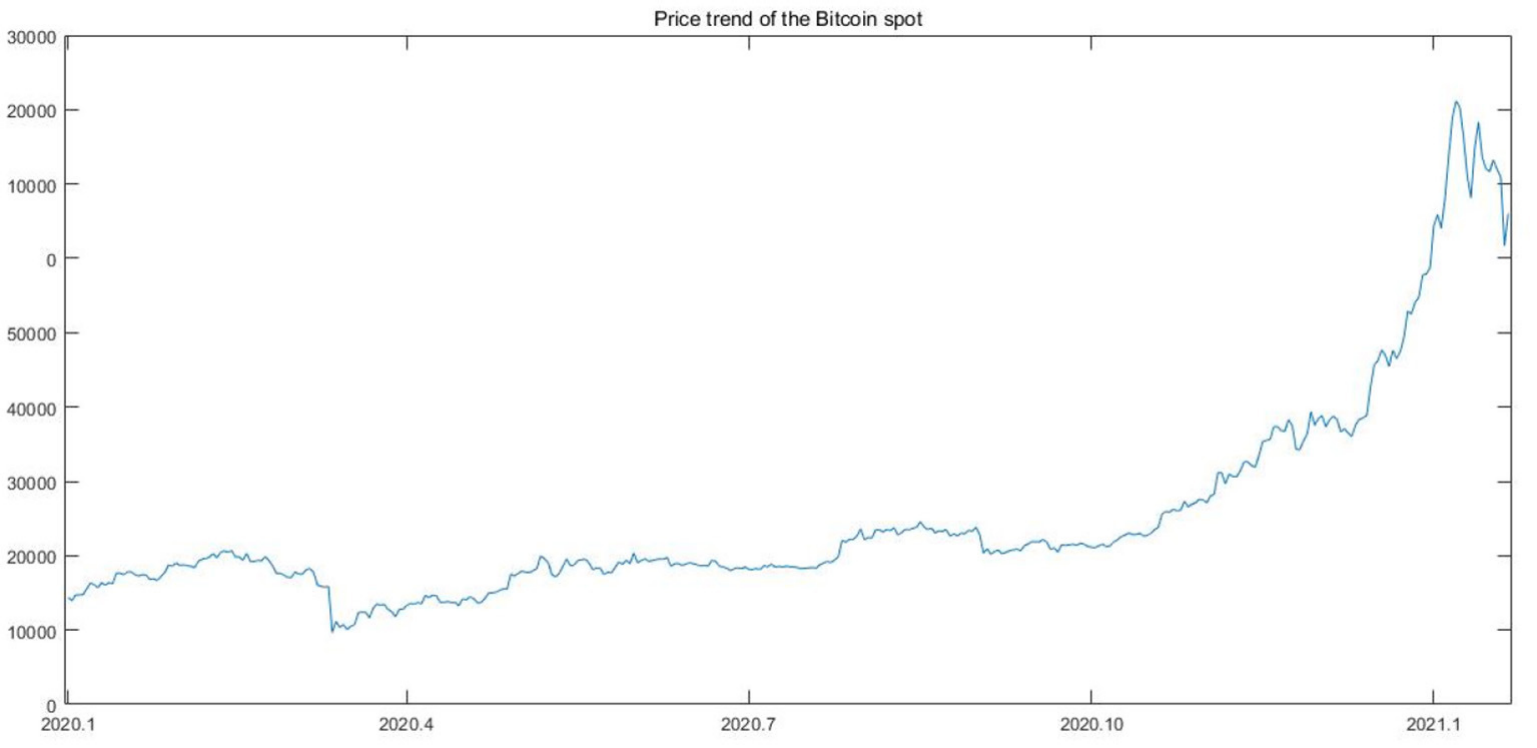
Price trend of the Bitcoin market from 2020 to 2021.

Bitcoin, as a type of digital cryptocurrency, raises great concerns with the help of promotion in technology in recent years (4). Bitcoin's unprecedented performance and volatility since its inception have sparked a great deal of concern from practitioners, regulators, and scholars since 2008. However, reviewing the development history of Bitcoin, it should be noted that the spot of Bitcoin was traded at a low price and trading volume before the end of 2017 when the Chicago Board Options Exchanges (CBOE) began to trade the Bitcoin futures. In 2018, the price of Bitcoin spot decreased rapidly before raising slightly in 2019. Then, in the first half of 2020, after the outbreak of Covid-19 and the consequent quantitative easing globally, the global financial markets witnessed the price of Bitcoin spot rise quickly to more than 40,000 dollars (See Figure 1). The drastic changes in Bitcoin price illustrate an interesting phenomenon that there is no difference between the general financial markets and the Bitcoin market.

With the rapid increase in Bitcoin spot price, there is no doubt that Bitcoin spot price poses significant risks for the investors in the Bitcoin market, which makes it an urgent issue for investors to seek a feasible way to reduce risks. A natural suggestion in the financial area is to consider the futures, namely, the Bitcoin futures, to reduce the risks brought by the sustained rise in the spot market. As a classic financial issue, using futures to hedge risks from the spot market has reached numerous achievements. For example, Chen et al. (5) investigated the effectiveness of carbon futures in hedging the risks in the carbon spot market; Zhao et al. (6) focused on the hedge strategies between crude oil spot and futures; Chan and Young (7) researched the copper futures and spot; Park and Switzer (8) estimated the optimal hedge ratio for stock and index futures. Benet (9) assessed the hedging effectiveness in the FX market and the like. These investigations all demonstrate an important reality in the financial area that the futures market is a perfect tool to hedge the risks from the corresponding spot market. The list is far from exhaustive and exemplifies how active the field remains. However, as an asset of the world's attention, it is worth noting that current investigations are not involved in Bitcoin hedging, and it is of great interest to us to explore whether the above suggestion is feasible.

In this paper, we focus our concerns on the hedging effectiveness between Bitcoin spot and futures. Considering the market correlation, volatility spillover exhibits a crucial role in the understanding of hedging effectiveness and portfolio management. Thus, in this paper, all the three aspects, i.e., correlation, volatility spillover, and hedging effectiveness between Bitcoin spot and futures are highlighted. And to the best of our knowledge, this paper makes the following contributions to the literature. First, it is the first attempt to investigate the dynamic correlation between Bitcoin spot and futures. Specifically, the VAR-DCC-GARCH model is adopted. The empirical results show that Bitcoin spot and futures markets have no difference compared with other spot and futures markets. The two markets are highly and positively correlated during our sample period. The results shed light on avoiding risk for investors. Second, it is the first time to investigate the volatility spillovers between Bitcoin spot and futures markets. In this paper, the VAR-BEKK-GARCH model is selected. The empirical results show that ARCH and GARCH effects do exist between Bitcoin spot and futures markets. In other words, there exists a bi-directional volatility spillover between the Bitcoin spot and futures. The results may deepen the understanding of information transmission between the two markets. Third, we focus our concerns on the issue of hedging effectiveness between Bitcoin spot and futures markets. Specifically, based on the results from the VAR-BEKK-GARCH model, we calculate a dynamic hedge ratio and the related hedging effectiveness. The results show that the value of hedging effectiveness (HE) is equal to 0.6446, which indicates that Bitcoin futures can be used to hedge the risks from the Bitcoin spot. The results directly benefit the investors in the Bitcoin market. Fourth, for the sake of guaranteeing the rigor of an academic research paper, we implement robustness checks. We especially evaluate the hedging effectiveness of Bitcoin futures to Bitcoin spot after the outbreak of the Covid-19 pandemic. The results of the robustness checks show that the hedging power of the Bitcoin futures improved, as the HE value is equal to 0.6778, which is larger than the case of the full sample. To sum up, all the results indicate that the increasing Bitcoin spot market risks can be hedged by the Bitcoin futures market. Finally, we make in-depth studies. On the one hand, we explore whether the traditional futures market can be used to hedge the risks of the Bitcoin spot market. Specifically, we chose the gold futures as gold is regarded as a safe haven asset. We re-do the VAR-BEKK-GARCH estimations, and the results show that the gold futures market can also be used to hedge the risks from the novel Bitcoin spot market despite the HE value being much smaller compared to the Bitcoin futures market. On the other hand, we control other cryptocurrencies to further investigate the hedging ability of the Bitcoin futures to the Bitcoin spot, the results prove that the even other factors which are closely related to the Bitcoin market, are controlled, the Bitcoin futures can still reduce the corresponding risks in the Bitcoin spot market.

This paper is organized as follows. Section A Brief Introduction About the Covid-19 briefly reviews the Covid-19. Section Related Literature shows the related literature. In section Data and Methodologies, we present the data and models used in this paper. Section Empirical Results shows the related empirical results. Robustness checks are shown in section Robustness Check. Further discussion is shown in section Discussion, and section Conclusion concludes the paper.

## A Brief Introduction About the Covid-19

Coronaviruses are responsible for several illnesses in both humans and animals. Coronaviruses cause zoonoses, such as the common cold, or severe respiratory diseases. Some coronaviruses are known to be circulating in different animal populations. However, once the viruses mutate, they can infect human beings. Covid-19 is the most recent one to make the jump to human infection (10).

Despite the disease brought by the virus being spreading undetected around the world, it was the Chinese government that first officially reported the disease, and, subsequently, the disease has become one of global concern. After systematic and scientific analysis, the International Committee on Taxonomy of Viruses officially named the coronavirus severe acute respiratory syndrome coronavirus 2 (SARS-CoV-2). Though the origin of the virus is unknown, it is widely accepted that after some necessary mutations, the virus can transmit from human to human with highly contagious, resulting in a deep binding between people and viruses. It is said that the Covid-19 has become the seventh documented pandemic after the black death, “Spanish” flu pandemic, The HIV/AIDS pandemic, the SARS and the “swine” flu pandemic, and the Avian flu, Ebola, and zika virus pandemics (11, 12).

Covid-19 has had a serious impact on several areas, such as, for example, the healthcare industry, international trade, economic and financial areas, etc. Given globalization, the modern economies are highly connected, which means the actual consequences of Covid-19 could be even worse. Covid-19 results in workers being absent from work due to illness or an increase in the risk of infection, which disrupts the normal production in countries. According to the International Labor Organization, working hours have significantly decreased during the Covid-19 pandemic. Consequently, the whole industrial chain received an immeasurable impact. In other words, the Covid-19 results in a global economic recession, which leads to expansionary monetary policy by central banks. Comprehensive coverage of the worldwide economic and financial effects of COVID-19 can be found in Fernandes (13).

## Related Literature

After the outbreak of the Covid-19, investigations about the impacts of the Covid-19 epidemic on the economy have become a research focus, and many scholars have studied the topic from diversified aspects. For example, Liu et al. (14) argued that Covid-19 provides considerable negative shock on the global stock market, and besides, these impacts vary between higher-income and lower-income countries. However, Liu et al. (15) held a different view; the authors showed that the Covid-19 has a statistically significant positive effect on the stock returns. Wu et al. (16), Lee et al. (17), and Hong et al. (18) focused on the impacts of Covid-19 on tourism and hospitality sectors as well as index returns, respectively. Lee and Chen (19) argued a non-linear effect of confirmed cases. Guerrieri et al. (20) had a comprehensive discussion of the consequences of different policies implemented during the Covid-19 pandemic. Particularly, Ozili and Arun (21) suggested that these policies ultimately affect stock prices. Baker et al. (22) adopted different information sources, i.e., volatility in the stock market, newspapers, and subjective uncertainty in business expectation surveys, to quantify the uncertainty during Covid-19; the authors argued that uncertainties increased enormously. Ramelli and Wagner (23) pointed out that the Covid-19 amplifies the economic and financial crisis. Zhang et al. (24) explored the impact of the Covid-19 from the macroeconomic level. The authors investigated the systemic risks of global financial markets and assessed the effects of policy interventions. Akhtaruzzaman et al. (25) focused on the linkages of the emerging Chinese market and the G7 countries during the Covid-19 pandemic. Specifically, the authors centered on the financial contagion and argued that the epidemic enhances the correlations. Sharif et al. (26) adopted several econometrics methods to analyze the Covid-19 and stated that the risks from Covid-19 have no difference with the economic crisis. Ji et al. (27) focused on the safe-haven roles during the Covid-19 pandemic and argued that gold and soybean commodity futures are still feasible while other classical assets do not. Salisu and Vo (28) linked the health news trend and stock market return. The authors pointed out that health news can significantly forecast the stock market during the epidemic. Based on the spillover index of Diebold and Yilmaz (29, 30), Wang et al. (31) pointed out that Covid-19 significantly influences the international financial markets. Qian et al. (32) noted that Covid-19 and the housing price are connected. Haroon and Rizvi (33) linked the news about Covid-19 and the volatility of equity markets, and So et al. (34) then showed that during the Covid-19 pandemic, the connections of the Hongkong financial market have become tighter. Baig et al. (35) centered on the liquidity and volatility in the equity markets in the US after the outbreak of the Covid-19 pandemic, while Heyden and Heyden (36) focused on the US and European stocks at an early stage of the epidemic. As an asset of the world focus, the cryptocurrency also became a research hotspot during the Covid-19 pandemic. For example, Corbet et al. (37) suggested that neither gold assets nor Bitcoin assets have been significantly connected with the Chinese stock market during Covid-19. Mariana et al. (38) made an attempt to explore whether the two largest cryptocurrencies, Bitcoin and Ethereum, can be safe-havens for stocks during Covid-19. Goodell and Goutte (39) linked the deaths caused by Covid-19 with the daily Bitcoin prices. Huang et al. (40) argued that Bitcoin contributes to the diversification benefits and other similar outcomes.

This paper is also related to a vast body of literature on the relationships between different financial markets. For example, several studies have focused on the analysis of the linkages between spot and futures commodity prices. Bessler and Covey (41) showed significant links between spot and futures prices for the US cattle markets. The findings of Silvapulle and Moosa (42) discovered the bi-directional non-linear Granger causalities between spot and futures prices in the WTI (West Texas Intermediate) crude oil market. Chen et al. (5) focused on the relationships between the carbon spot and futures. Other studies focused on hedging between markets. Hedging is also a focus in modern finance, attracting numerous studies. Time-varying variance and covariance play prominent roles. Existing studies mainly adopted the multi-variate GARCH models. For example, through the multi-variate GARCH model, Chang et al. (43, 44) made a deep investigation into hedging and portfolio performance. Furthermore, Chang et al. (45) argued that the constant correlation coefficient (CCC)-GARCH model can outperform other multi-variate GARCH models. However, Billio et al. (46) and Hung et al. (47) held a different, the authors suggested that switching to the GARCH model can significantly beat the CCC-GARCH model. Empirical evidence also shows that complex hedging strategies do not always empirically outperform naive hedging strategies [see, for example, Khalfaoui et al. (48) and Wang et al. (49)]. In addition, there also exists a few papers on hedging under alternative frameworks, for example, those by Sukcharoen and Leatham (50) and Chun et al. (51). Recently, researchers were beginning to explore hedging in the emerging market. For example, Jin et al. (52) explored the risk hedging in the carbon market by other financial markets through several multivariate GARCH models.

Despite the fact that the Bitcoin market is becoming an interesting market to test financial characteristics, i.e., the bubbles and market efficiency (53, 54), the connections between Bitcoin spot and futures have not yet been studied thoroughly. Thus, in this paper, we pay attention to the Bitcoin market, especially to Bitcoin spot and futures markets, to fulfill the potential research gap. Specifically, in this paper, we investigate the dynamic correlation, volatility spillover, and hedging effectiveness between the Bitcoin spot and futures markets. The results in this paper may benefit the understanding of the Bitcoin market. And more importantly, the results in this paper may guide the investors to reduce the risks in the Bitcoin spot market by the Bitcoin futures.

## Data and Methodologies

### Data

In this paper, we collected our dataset on daily Bitcoin spot and futures from Investing (https://cn.investing.com/), which is a commonly used website to collect financial time series. Considering the quality of data on Bitcoin futures, we do not pick the data from the very beginning of Bitcoin futures. Specifically, in this paper, we collect our data from Dec 11, 2017, to Jan 22, 2021. Some basic descriptive statistics of the Bitcoin spot and futures returns are shown in the following Table 1.

**Table 1:** Basic descriptive statistics of Bitcoin spot and futures.

	**Bitcoin spot**	**Bitcoin futures**
Min	−0.3918	−0.23349
Max	0.1723	0.2545
Std. dev	0.0420	0.0442
Mean	0.0011	0.0018
Skewness	−0.7102	0.0585
Kurtosis	9.6228	4.2951

**Figure 2:**
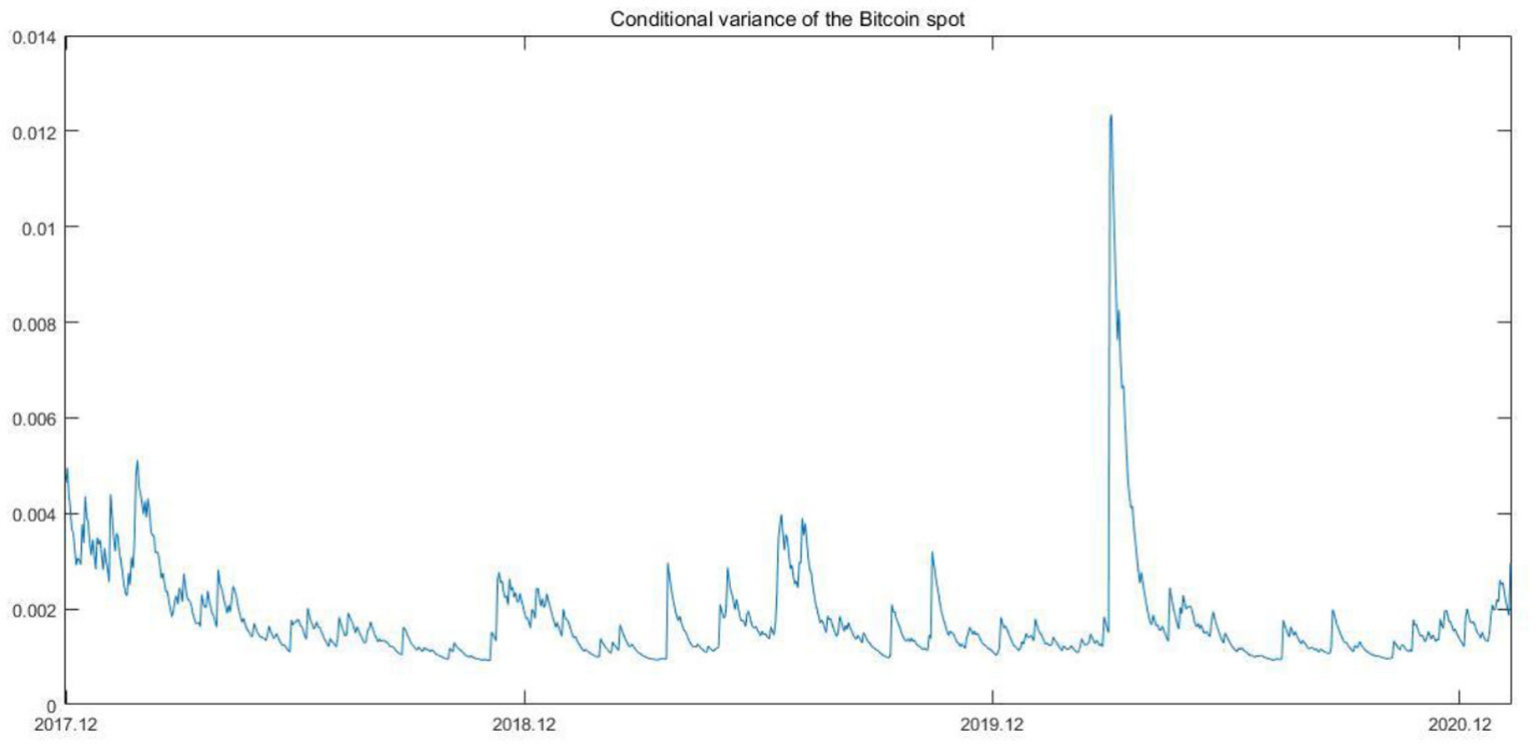
Conditional variance of the Bitcoin spot.

**Figure 3:**
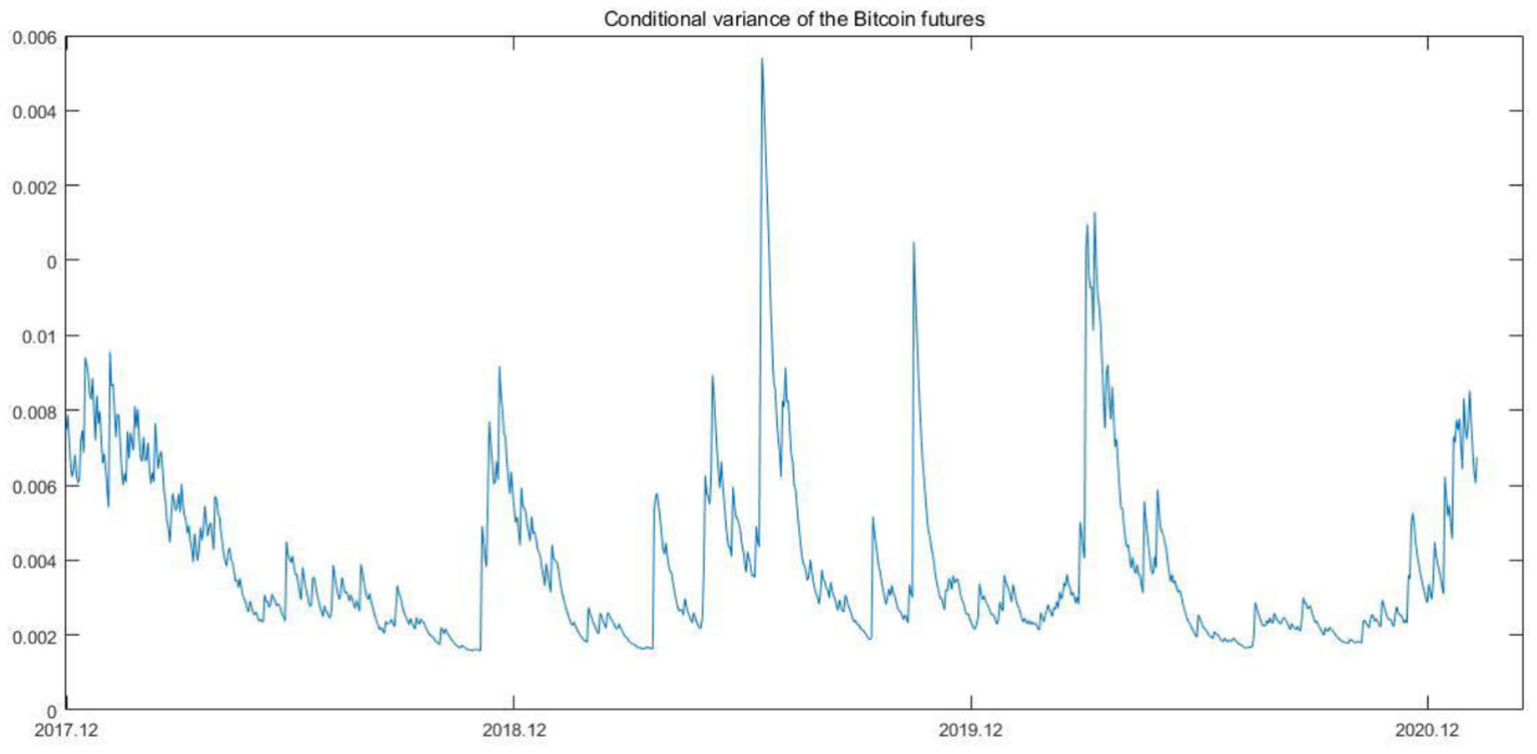
Conditional variance of the Bitcoin futures.

As shown in Table 1, on the one hand, the difference between the minimized value and maximized value is drastic, which may represent that the prices fluctuate wildly; on the other hand, the mean values of Bitcoin spot and future are positive with the magnitude of 0.0011 and 0.0018, respectively, which indicates that the prices show a sustained increasing trend during our sample period. Considering the daily frequency, such increasing is huge. The kurtosis of Bitcoin spot and futures returns exceeds three, and the skewness of these returns is not equal to zero. Such characteristics indicate that Bitcoin spot and futures return series have the characteristics of most financial time series, namely, the series are not normally distributed and have fat tails. In most of cases, the univariate GARCH model is apparently successful in processing the volatility of the financial time series. Besides, according to previous studies (55, 56), GARCH (1,1) is enough to summarize the volatility. Thus, in this section, we implement the GARCH (1,1) process to depict the fluctuation of the two series. The conditional variances are shown in Figures 2, 3.

As shown in the above two figures, the two series for conditional variances have almost the same tendency. The Bitcoin futures seems to be more violate than the Bitcoin spot except for the time when Covid-19 broke out in the first half of 2020. In addition, it is obvious that when Covid-19 spread globally, the conditional variance reached the maximized value, and thereafter, the condition variances show an upward tendency, which is not consistent with the trend of conditional variance after the occurrence of outliers in the pre-Covid-19 times. The upward trend reflects the increasing risks in the Bitcoin market to some extent.

### VAR Model

Before estimating the parameters for the variance equation in the multi-variate GARCH models, for example, the DCC-GARCH or the BEKK-GARCH model, it is essential to get the residuals of the return series from the mean equation. Classical estimation methods to get the residuals seem to suppose that the mean equation in the GARCH model is based on one constant. However, the assumption seems unreliable in some cases when the transaction costs are too important to ignore. Thus, estimation methods that can deal with the deficiency are desperately needed. In the financial area, it is widely accepted that the VAR model can effectively reduce the influence of transaction costs in estimating and predicting asset return (57, 58). Therefore, in this paper, we adopt the widely used VAR model to update the mean equation in order to better fit the Bitcoin spot and futures return and get the residuals. In other words, in this paper, the residuals for multivariate GARCH models are based on the VAR model. The VAR model can be summarized as follows

(1) $$\begin{array}{r} R_{t}=C+\sum_{i=1}^{p}R_{t-i}+\varepsilon_{t} \end{array}$$

(2) $$\begin{array}{r} \varepsilon_{t}\sim N\left(0,H_{t}\right) \end{array}$$

where *R*_*t*_ is a vector containing two dimensions, i.e., the Bitcoin spot and the Bitcoin futures, *C* is the constant, *p* refers to the lag length of the VAR model, and ε_*t*_ is the residual. As for *H*_*t*_, it refers to the conditional-variance-covariance matrix of ε_*t*_.

### DCC-GARCH Model

The DCC and CCC model specification are closely connected. Despite the variance-covariance matrix being time varying, the CCC model assumes that the correlation between assets is a constant. However, such an assumption may not be appropriate for economic variables in some cases. Thus, Engle (59) relaxed the assumption on constant correlation coefficient to the dynamic correlation coefficient and put forward the DCC model. Thereafter, the DCC specification is adopted by numerous investigations to depict the correlation between assets. The DCC model can be summarized by the following Equations (3–6).

(3) $$\begin{array}{r} H_{t}=D_{t}R_{t}D_{t} \end{array}$$

(4) $$\begin{array}{r} R_{t}=Q_{t}^{*-1}Q_{t}Q_{t}^{*-1} \end{array}$$

(5) $$\begin{array}{r} D_{t}=diag\left(\sqrt{h_{11,t},}\ldots ,\sqrt{h_{NN,t},}\right) \end{array}$$

(6) $$\begin{array}{r} Q_{t}=\left(1-\alpha -\beta \right)\bar{Q}+\alpha Q_{t-1}+\beta \delta_{i,t-1}\delta_{j,t-1} \end{array}$$

In the above expressions, *R*_*t*_ is a symmetric conditional correlation matrix, *D*_*t*_ is a diagonal matrix containing standard deviations estimated by the univariate GARCH model, and *h*_*ii,t*_(*i* = 1, …, *N*) represents the diagonal elements of *H*_*t*_, $\overset{-}{Q}$ represents the unconditional correlation matrixes. Besides, the model requires both α and β in Equation (6) to be non-negative and satisfy a condition that α + β < 1. $Q_{t}^{*}$ is a diagonal matrix with the squared root of the ith diagonal element of *Q*_*t*_ on its corresponding location.

### BEKK-GACRH Model

Despite the numerous achievements in the application of the DCC model to depict the dynamic correlation between assets, the direction of the correlation cannot be inferred from the DCC-GARCH model. In other words, the existence and direction of volatility spillover from one asset to another asset cannot be identified. Thus, in this paper, we make an in-depth investigation to explore this issue between Bitcoin spot and futures. According to Chen et al. (5), the BEKK model is suitable to solve the issues of volatility spillover. Thus, in this paper, the BEKK model is also selected. The BEKK-GARCH model is represented by the following Equation (7),

(7) $$\begin{array}{r} H_{t}=CC^{\prime }+BH_{t-1}B^{\prime }+A\varepsilon_{t-1}{\varepsilon_{t-1}}^{\prime }A^{\prime } \end{array}$$

where *H*_*t*_ is the conditional variance-covariance matrix, the matrix of B is the parameter matrix for conditional variance, representing the relationship of variances between assets, while the matrix of A is the parameter matrix for the residual achieved by the mean equation. According to econometric theories, the matrix of A captures the ARCH effect, while B reflects the GARCH effect.

## Empirical Results

### Dynamic Correlation

As mentioned in the above section, it is essential to identify the lag length for the VAR model before estimating the dynamic correlation and volatility spillover. In this part, we report the lag length selection process in the following Table 2.

**Table 2:** Lag length selection for the VAR model.

**Lag**	**LR**	**FPE**	**AIC**	**SC**	**HQ**
1	227.9593	1.04e-06	−8.0966	−8.0663^*^	−8.0850
2	17.3786	1.03e-06	−8.1064	−8.0559	−8.0872
3	23.3818^*^	1.02e-06^*^	−8.1225^*^	−8.0518	−8.0956^*^
4	2.7452	1.02e-06	−8.1171	−8.0262	−8.0825

*LR means the sequentially modified LR test statistic; FPE is the Final prediction error; AIC represents the Akaike information criterion; SC refers to the Schwarz information criterion; HQ is the Hannan-Quinn information criterion. e-06 means 10^∧^ (−6) while*

* *indicates the lag order selected by the criterion*.

**Figure 4:**
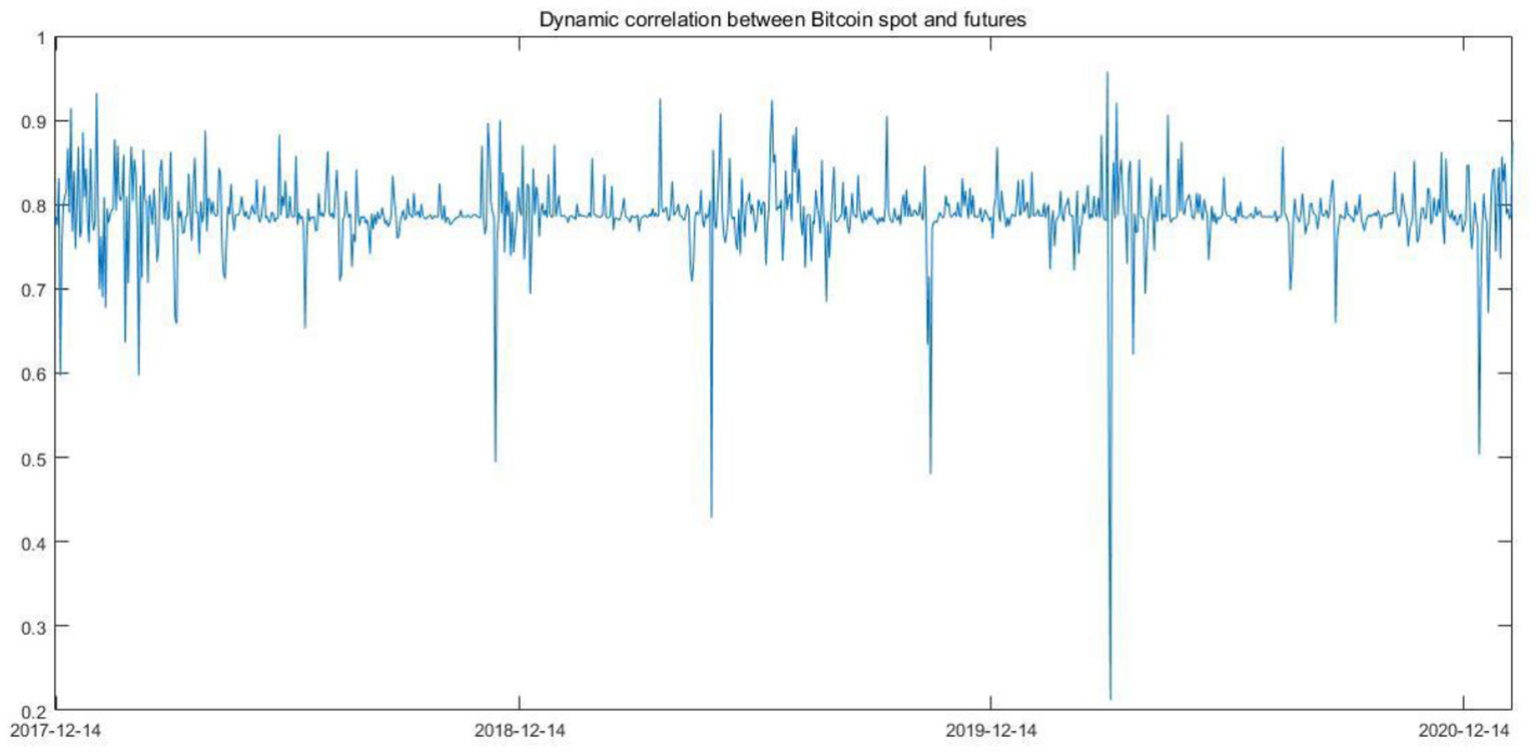
Dynamic correlation between Bitcoin spot and futures.

As shown by the above Table 2, according to FPE, AIC, and HQ criteria, we set the lag length in the VAR model to be 3, and besides, as mentioned above, GARCH (1,1) is enough to summarize to variance equation in the GARCH models. Therefore, in this paper, we combine the VAR model and the DCC-GARCH model to form the VAR-DCC-GARCH (3,1,1) model to depict the dynamic correlation between Bitcoin spot and futures. Basic information of the dynamic correlation is shown in the following Figure 4 and Table 3.

**Table 3:** Descriptive statistics of the dynamic correlation.

	**Mean**	**Max**	**Min**
Value	0.7886	09580	0.2120

As shown in Figure 4, the Bitcoin spot and futures are highly correlated. From Table 3, it is clear that the mean value of the dynamic correlation series is equal to 0.7886. However, extreme values also exist. For example, before the Covid-19 global outbreak in March 2020, the correlation showed an upward trend and reached the maximized value with the magnitude of 0.9580, and upon the global outbreak of Covid-19 in March 2020, the correlation between the Bitcoin spot and futures reached the minimized value with the magnitude of 0.2120.

### Volatility Spillover

It is essential to determine the two parameters in the variance equation of the BEKK-GARCH model. According to previous studies, (1,1) is widely used as the parameters and is proved to be sufficient to capture the volatility spillovers between assets. Thus, considering the lag length in the VAR model is equal to 3, in this section, we construct the VAR-BEKK-GARCH (3,1,1) model. The estimation results for the variance equation regarding volatility spillover are as follows in Table 4.

**Table 4:** Estimation of VAR-BEKK-GARCH (3,1,1) for volatility spillover.

**Panel A: Estimation results**			
	**Coeff**	**Std Error**	**T-Stat**
C(1,1)	0.0193	0.0017	11.5710***
C(2,1)	0.0166	0.0022	7.6808***
C(2,2)	0.0000	0.0016	0.0000
A(1,1)	−0.0710	0.0615	−1.1539
A(1,2)	−0.5215	0.0626	−8.3368***
A(2,1)	−0.1200	0.0637	−1.8823*
A(2,2)	0.0569	0.0507	1.1219
B(1,1)	1.4054	0.0450	31.2593***
B(1,2)	0.7036	0.0476	14.7782***
B(2,1)	−0.7407	0.0664	−11.1519***
B(2,2)	0.1452	0.0602	2.4115**
**Panel B: Wald test**			**Wald Test**
A(1,2) = B(1,2)			268.8633***
A(2,1) = A(2,1)			126.3361***
A(1,2) = B(1,2) = A(2,1) = A(2,1)			269.6281***

A(i,j), B(i,j), and C(i,j) are elements for matrix A, B, and C, respectively. *, **, and *** denotes significance at 10, 5, and 1% level, respectively.

According to the parameters depicted in Table 4, we can obtain the following conclusions that there exists a bi-directional arch type and GARCH type volatility spillovers between Bitcoin spot and futures, as A(1,2), A(2,1), B(1,2), and B(2,1) are significant. And besides, the results also reflect a fact that Bitcoin spot and futures all show volatility clustering. Furthermore, we conduct the Wald-test for the interested parameters, and the results support the above conclusions that the volatility of Bitcoin futures can impact that of Bitcoin spot, vice versa. The bi-directional existence of volatility spillover between Bitcoin spot and futures may be explained by the following reasons. Bitcoin spot and futures markets are affected by the same economic and technology factors, for example, micro-foundation, market investors, and information sources, are almost the same for Bitcoin spot and futures. These common factors will lead to volatility linkages by altering expectations and thereby affecting asset demand or cross-market hedging.

### Hedging Effective

The above two sections demonstrate two important facts in the Bitcoin market that Bitcoin spot and futures are highly correlated and there exists volatility spillover. In this part, we make an in-depth investigation to explore the application of volatility spillover. Specifically, we explore the issue of whether the Bitcoin futures is feasible to hedge risks from the Bitcoin spot based on the results of VAR-BEKK-GARCH.

Based on the modern portfolio theory, Johnson (60) put forward the well-known minimum variance hedge ratio model for minimizing the risk associated with a certain portfolio. In this model, it is assumed that asset j can be used as a hedge for another asset i. The return of the hedged portfolio can be written as follows in the following Equation (8),

(8) $$\begin{array}{r} R_{H,t}=R_{s,t}-\gamma_{f,t}R_{f,t} \end{array}$$

where *R*_*H,t*_ represents the return of the hedged portfolio, γ_*f,t*_ is the hedge ratio, *R*_*s,t*_, and *R*_*f,t*_ represent the return of Bitcoin spot and Bitcoin futures, respectively. According to Johnson (60), Baillie and Kroner, the optimal hedge ratio $r_{f,t}^{*}$ in Equation (8) can be achieved by the following Equation (9),

(9) $$\begin{array}{r} r_{f,t}^{*}=\frac{h_{t}^{st}}{h_{t}^{ff}} \end{array}$$

where $h_{t}^{st}$ is the co-variance between Bitcoin spot and futures, $h_{t}^{ff}$ is the conditional variance of the Bitcoin futures. The two parameters, i.e., $h_{t}^{st}$ and $h_{t}^{ff}$, are calculated by the multivariate GARCH model, i.e., VAR-BEKK-GARCH. In order to evaluate the effectiveness of the hedging strategy, we further introduce an indicator, i.e., hedging effectiveness (HE), which is widely used by previous studies. The indicator of HE is calculated by the following Equation (11).

(10) $$\begin{array}{r} HE=\frac{\left(var_{unhedged}-var_{hedged}\right)}{var_{unhedged}} \end{array}$$

where *var*_*hedged*_ represents the variance of the hedged portfolio and can be obtained through the time series of *R*_*H,t*_. As for the *var*_*unhedged*_, the variable refers to the variance of the unhedged portfolio. Commonly, it refers to the variance of spot returns.

**Figure 5:**
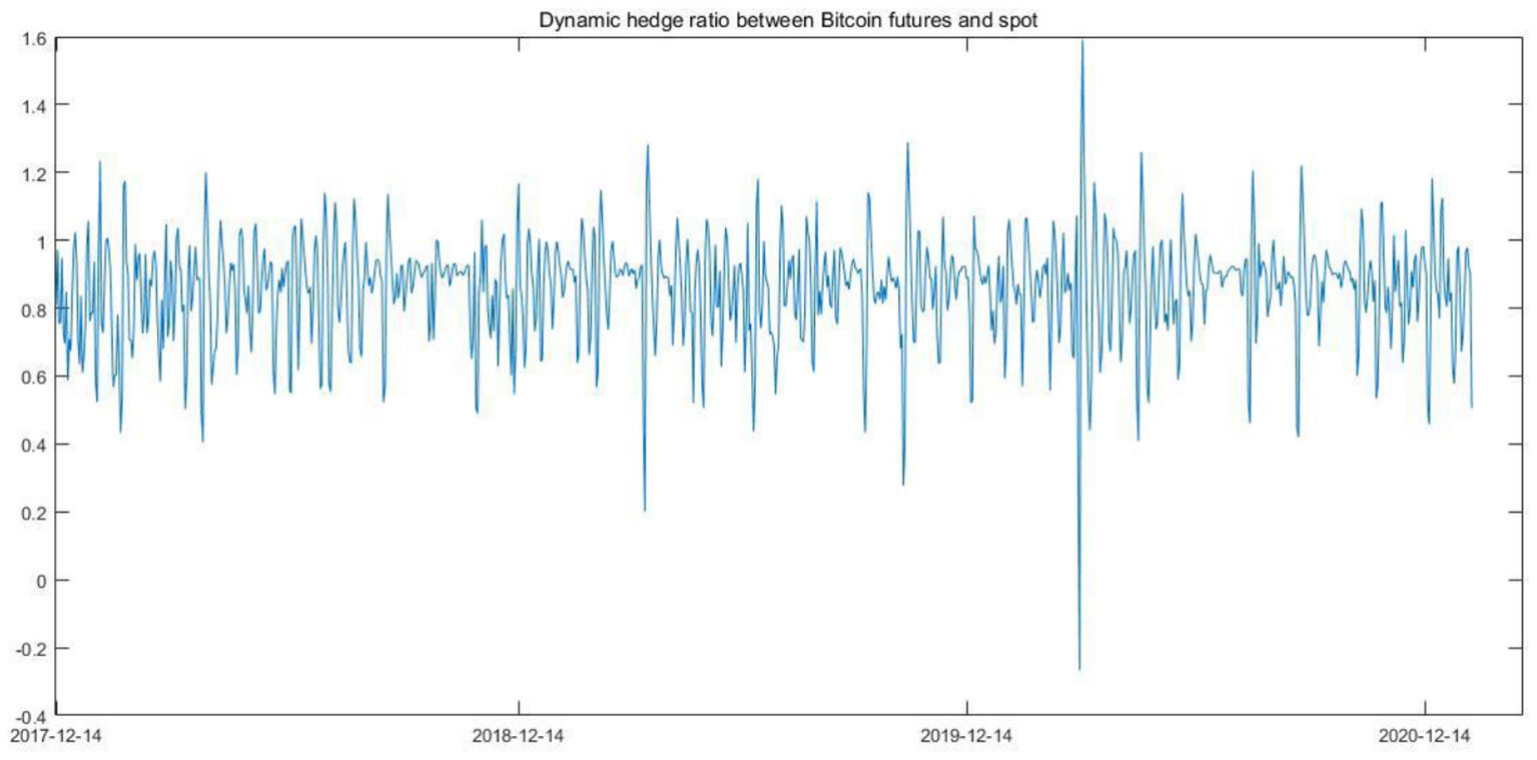
Dynamic hedge ratio between Bitcoin futures and spot.

The HE value is smaller than 1. If HE is negative, it means that investors do not need to hedge risks in the spot market by the futures market. On the contrary, a positive HE means that futures markets indeed reduce the risk in the spot market. Moreover, the closer the HE gets to 1, the better risk reduction of Bitcoin futures to Bitcoin spot. We show the HE value and the basic information of the optimal hedge ratio in the following Table 5 and Figure 5, respectively.

**Table 5:** Basic information of the dynamic hedging strategy.

**Hedging characteristics**	**Value**
Average Hedge ratio	0.8572
Min	1.5899
Max	−0.2672
HE value	0.6446

As shown in Table 5, Bitcoin futures can surely be used as a hedge to reduce the risks from the Bitcoin spot market as the HE value is equal to a positive 0.6446. The HE is equal to 0.6446, indicating that around 64.46% of the price return variance of the Bitcoin spot can be effectively hedged by taking a short position in Bitcoin futures. The optimal hedge ratio for Bitcoin futures is 0.8572. This means that a unit of the long position in the Bitcoin spot can be hedged by shorting 0.8572 of carbon futures on average.

## Robustness Check

**Figure 6:**
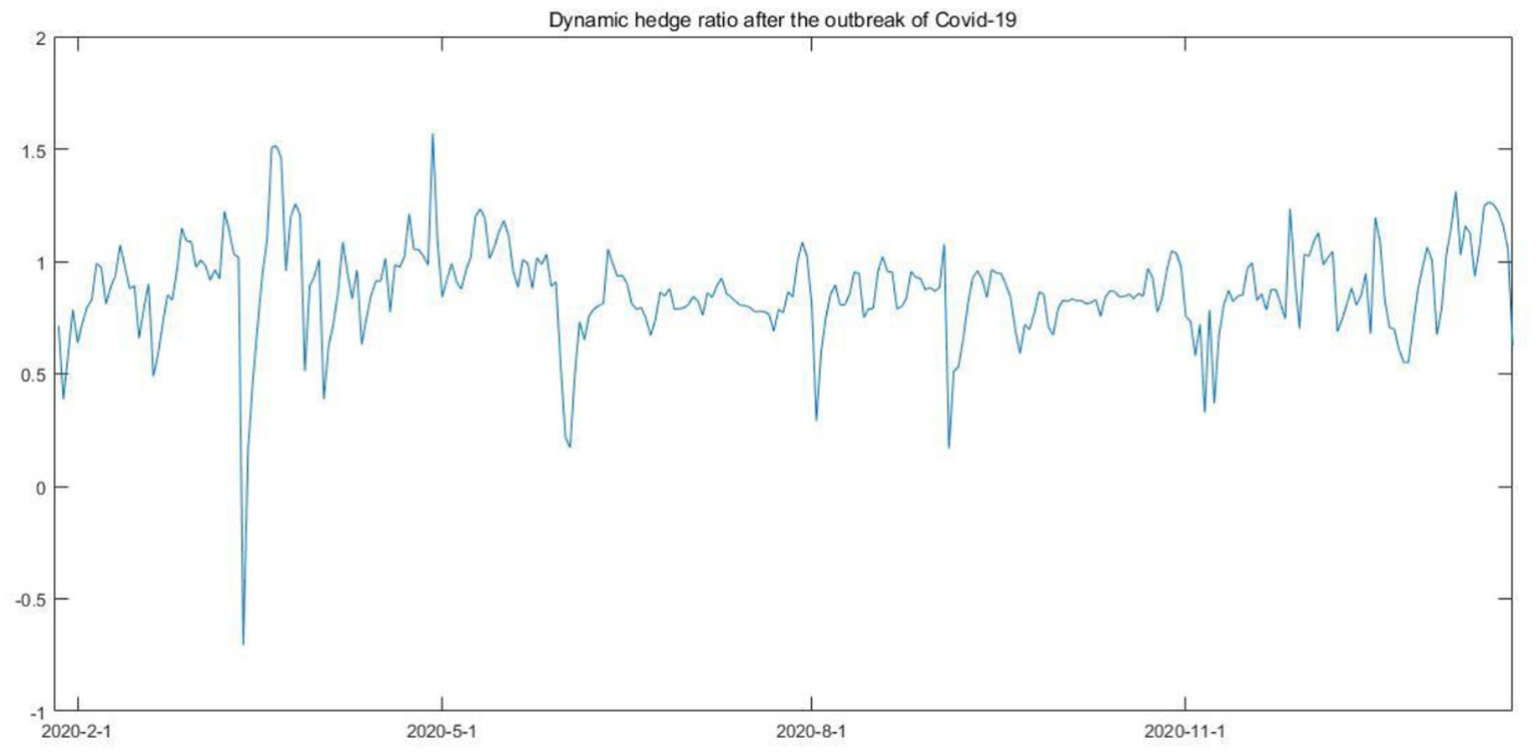
Dynamic hedge ratio after the outbreak of Covid-19.

The above illustration proves an important fact that futures, especially the Bitcoin futures, can be used as a hedge to reduce risks in the Bitcoin spot market. However, for the sake to guarantee the rigor of an academic research paper, it is essential to verify the conclusion by updating the data of futures. Thus, in this section, we alter the time period for empirical investigations. In this section, we choose a particular sample from the very beginning of the Covid-19 to the end of our sample to make an in-depth study on the hedging power of Bitcoin futures to Bitcoin spot. Specifically, we selected the time period from Jan 24, 2020, to Jan 22, 2021, as our sample and re-did the VAR-BEKK-GARCH model for this sample. The results for volatility spillover, HE value, and dynamic hedge ratio are shown in Table 6 and Figure 6, respectively.

**Table 6:** Estimation results of VAR-BEK-GARCH after the outbreak of Covid-19.

**Panel A: Estimation results**			
	**Coeff**	**Std Error**	**T-Stat**
C(1,1)	0.0100	0.0024	4.1181***
C(2,1)	0.0183	0.0026	7.0961***
C(2,2)	−0.0000	0.0032	−0.0000
A(1,1)	−0.1036	0.1613	−0.6426
A(1,2)	−0.8976	0.1425	−6.2992***
A(2,1)	0.5827	0.1370	4.2521***
A(2,2)	1.0287	0.1572	6.5457***
B(1,1)	0.8437	0.0703	11.9989***
B(1,2)	0.1573	0.0791	1.9890**
B(2,1)	0.0275	0.0847	0.3240
B(2,2)	0.6105	0.0870	7.0179***
**Panel B: Wald test**			**Wald test**
A(1,2) = B(1,2)			39.7349***
A(2,1) = A(2,1)			31.8814***
A(1,2) = B(1,2) = A(2,1) = A(2,1)			79.7450***
**Panel C: Basic information of the dynamic hedging**			**Value**
Average Hedge ratio			0.8689
Min			1.5691
Max			−0.7074
HE value			0.6778

A(i,j), B(i,j), and C(i,j) are elements for matrix A, B and C, respectively. **, and *** denotes significance at 10, 5 and 1% level, respectively.

As shown in Table 6, the bi-directional volatility spillover between Bitcoin spot and futures does not change as the interested parameters are significant. More importantly, the results indicate that after the outbreak of Covid-19, compared with the case for the full sample, the hedging power of the Bitcoin futures improved, as HE increases from 0.6446 to 0.6778. However, the results also indicate another fact in the Bitcoin market, Bitcoin futures have always been a good hedge against bitcoin spot risk, as the increase of HE value is quite limited.

## Discussion

The global plague, Covid-19, is still spreading, resulting in quantitative easing (QE), which to some extent pushes up Bitcoin prices. In this paper, we make in-depth investigations to explore whether the traditional assets can be used to hedge risks in the Bitcoin spot market. As Ji et al. (27) noted that the gold asset is a commonly used safe-haven asset, in this paper, we thus replace the Bitcoin futures with gold futures to re-investigate the functions of futures in hedging Bitcoin spot market risks. Specifically, we collect our data on gold futures from Investing (https://cn.investing.com/). As our hedging strategy is based on the VAR-BEKK-GARCH model, we re-do the estimations of the VAR-BEKK-GARCH model. The estimation results of the VAR-BEKK are also reported in this paper, see the following Table 7. And the corresponding statistics of the dynamic hedge ratio and the HE value are reported in Table 8.

**Table 7:** Volatility spillover estimated from VAR-BEKK-GARCH.

**Panel A: Estimation results**			
	**Coeff**	**Std Error**	**T-Stat**
C(1,1)	0.0190	0.0034	5.5366***
C(2,1)	−0.0047	0.0010	−4.7829***
C(2,2)	−0.0000	0.0024	−0.0000
A(1,1)	0.2336	0.0446	5.2419***
A(1,2)	−0.0476	0.0127	−3.7364***
A(2,1)	0.1774	0.1304	1.3605
A(2,2)	0.4360	0.0506	8.6093***
B(1,1)	0.8691	0.0476	18.2750***
B(1,2)	0.1142	0.0126	9.0415***
B(2,1)	−0.4735	0.1576	−3.0053***
B(2,2)	0.7086	0.0511	13.8699***
**Panel B: Wald test**			**Wald Test**
A(1,2) = B(1,2)			90.753734***
A(2,1) = A(2,1)			9.652930***
A(1,2) = B(1,2) = A(2,1) = A(2,1)			98.141248***

A(i,j), B(i,j), and C(i,j) are elements for matrix A, B and C, respectively. *** denotes significance at 10%, 5% and 1% level, respectively.

**Table 8:** Basic information of the dynamic hedging strategy for further discussion.

**Hedging characteristics**	**Value**
Average Hedge ratio	0.4301
Min	−2.1427
Max	1.7865
HE value	0.0326

From Table 7, it is clear that the ARCH type volatility spillover from Bitcoin spot to gold futures does not exist as A(2,1) is insignificant. However, the significance of the other parameters and the Wald test indicate that there exists a bi-directional volatility spillover between the Bitcoin spot and the gold futures markets. Besides, based on all these results for the VAR-BEKK-GARCH model, we calculate the HE value, and the results show that the gold futures market can be used as a hedge to reduce risks in the Bitcoin spot market as the HE value is larger than 0 (See Table 8). However, the results in Table 8 also reflect a fact that the traditional gold futures may not perform well in reducing the Bitcoin spot risks compared with the Bitcoin futures market as the HE is much smaller.

Existing study regarding the Bitcoin market shows that other cryptocurrencies are non-negligible factors to the Bitcoin market (61). Thus, in this paper, based on previous conclusions, other two empirical investigations are implemented. First, we control other cryptocurrencies as Katsiampa et al. (61) do. Second, we control one cryptocurrency in particular, i.e., Neo, as Katsiampa et al. (61) noted that cryptocurrency investors pay the most attention to news relating to Neo. In this paper, data on other cryptocurrencies, i.e., Ethereum (ETH), Litecoin (LTC), Dash (DASH), Ethereum Classic (ETC), Monero (XMR), Neo (NEO), and OmiseGO (OMG), are downloaded freely from Investing (https://cn.investing.com/). We re-do the above estimations for hedging effectiveness, and the detailed results of the two empirical investigations are shown in the following Table 9.

**Table 9:** Hedging effectiveness of the Bitcoin futures to the Bitcoin spot after controlling other cryptocurrencies.

**Hedging characteristics**	**Panel A**	**Panel B**
Average Hedge ratio	0.8218	0.8431
Min	−0.1027	−0.3597
Max	1.1425	1.2166
HE value	0.6670	0.6744

*Panel A is the case for controlling all the other cryptocurrencies, i.e., Ethereum (ETH), Litecoin (LTC), Dash (DASH), Ethereum Classic (ETC), Monero (XMR), Neo (NEO), and OmiseGO (OMG); Panel B refers to the case to control the Neo (NEO). The lag length of the VAR model for Panel A is equal to 1, thus, a VAR-BEKK-GARCH (1,1,1) model is estimated. The lag length of the VAR model for Panel B is equal to 3, thus, a VAR-BEKK-GARCH (3,1,1) is implemented*.

As shown in Table 9, it is clear that after controlling other cryptocurrencies, the Bitcoin futures can still be used to hedge the Bitcoin spot risks as the HE is still positive. The empirical results in this section further demonstrate that our conclusion is robust and thus can be used in Bitcoin investing.

## Conclusion

The Covid-19 outbreak has made Bitcoin a focus of research. The novelty of this paper lies in the analysis of relationships between the Bitcoin spot and futures markets. Specifically, in this paper, we investigate the dynamic correlation by the VAR-DCC-GARCH model and the volatility spillover by the VAR-BEKK-GARCH model. And based on the estimation results of the VAR-BEKK-GARCH model, one hedging strategy is put forward and assessed. The empirical results indicate that the Bitcoin spot and futures have no difference from traditional markets. Bitcoin spot and futures are highly correlated. The estimation results for the VAR-BEKK-GARCH model show that volatility spillover exists and is bi-directional. The hedging strategy based on the VAR-BEKK-GARCH model shows that the Bitcoin futures can be used as a hedge to reduce the risks in the Bitcoin spot market. Moreover, we especially focus on the post-Covid-19 period for robustness checks, the results show that the hedging ability increased as the HE value increased from 0.6446 to 0.6778. Finally, we further explore whether the traditional gold asset can be used to hedge the Bitcoin spot market. The results show that gold futures perform worse compared with the Bitcoin futures.

However, deficiencies also exist in this paper. For example, methods to depict the dynamic correlation or the volatility spillover are abundant. In this paper, only the VAR-DCC-GACH and the VAR-BEKK-GARCH are selected. Choosing alternative methods, for example, the GAS model (62) etc., to investigate the relationships inside the Bitcoin market is desperately needed to form a more comprehensive picture; At the same time, as Katsiampa et al. (61) noted that high-frequency volatility co-movements in cryptocurrency markets surely exist and adopting high-frequency data to further explore the dynamic correlation and volatility spillover is thus also desperately needed; As Xu (63) noted that energy markets spill to the emerging carbon market, it may also be an interesting topic to discuss the spillovers from energy markets to the emerging Bitcoin market.

## Data Availability Statement

The raw data supporting the conclusions of this article will be made available by the authors, without undue reservation.

## Author Contributions

YZ: conceptualization, methodology, software, data curation, and writing-original draft preparation. PZ: data curation and writing- reviewing and editing. YX: writing-original draft preparation and funding. All authors contributed to the article and approved the submitted version.

## Conflict of Interest

The authors declare that the research was conducted in the absence of any commercial or financial relationships that could be construed as a potential conflict of interest.

## Publisher's Note

All claims expressed in this article are solely those of the authors and do not necessarily represent those of their affiliated organizations, or those of the publisher, the editors and the reviewers. Any product that may be evaluated in this article, or claim that may be made by its manufacturer, is not guaranteed or endorsed by the publisher.
